# The Role of Chlorine in the Formation and Development of Tap Water Biofilms under Different Flow Regimes

**DOI:** 10.3390/microorganisms11112680

**Published:** 2023-10-31

**Authors:** Erifyli Tsagkari, William Sloan

**Affiliations:** School of Engineering, University of Glasgow, Glasgow G12 8QQ, UK; william.sloan@glasgow.ac.uk

**Keywords:** biofilm, shear stress, chlorine

## Abstract

Water companies make efforts to reduce the risk of microbial contamination in drinking water. A widely used strategy is to introduce chlorine into the drinking water distribution system (DWDS). A subtle potential risk is that non-lethal chlorine residuals may select for chlorine resistant species in the biofilms that reside in DWDS. Here, we quantify the thickness, density, and coverage of naturally occurring multi-species biofilms grown on slides in tap water with and without chlorine, using fluorescence microscopy. We then place the slides in an annular rotating reactor and expose them to fluid-wall shears, which are redolent of those on pipe walls in DWDS. We found that biofilms in chlorine experiment were thicker, denser and with higher coverage than in non-chlorine conditions under all flow regimes and during incubation. This suggests that the formation and development of biofilms was promoted by chlorine. Surprisingly, for both chlorinated and non-chlorinated conditions, biofilm thickness, density and coverage were all positively correlated with shear stress. More differences were detected in biofilms under the different flow regimes in non-chlorine than in chlorine experiments. This suggests a more robust biofilm under chlorine conditions. While this might imply less mobilization of biofilms in high shear events in pipe networks, it might also provide refuge from chlorine residuals for pathogens.

## 1. Introduction

Water emerging from taps in DWDS is routinely tested for the presence of coliform bacteria that might indicate microbial contamination [1]. There is evidence that even in well-managed DWDS, the incidents of water failing these tests are increasing [1,2] and that contaminated drinking water can cause serious health problems that are sufficient and widespread enough to affect economies [3,4]. In many countries, including the United Kingdom, chlorine disinfectant is added as the final stage of treatment at various locations through the DWDS to maintain low-level residual chlorine in the pipes to eliminate bacterial growth [5,6]. However, biofilms still grow on pipe walls in DWDS [7]. Some countries rely on the integrity of their pipe networks and prohibit chlorine residuals due to concerns over health risks from disinfection by-products as well as taste, colour, and odour changes in tap water [8]. Given that microbial regrowth occurs in biofilms in both chlorinated and non-chlorinated systems, the efficacy and health trade-offs of maintaining chlorine residuals in networks has become a topic of debate. To inform this debate, it is important to gather information on how the drinking water microbial diversity, pathogen abundance, bacterial phenotypes and the properties of multi-species biofilms are affected by chlorine residuals.

Organisms within biofilms can survive longer and may have greater chlorine resistance than those in the water phase [9,10]. This is due to protection that is conveyed by the EPS. Biofilms act as a reservoir of microorganisms (including pathogens) adhering to the pipe walls coated by EPS, which conveys mechanical stability [11]. The effect of chorine residuals on single-species biofilms has already been studied. Thus, chlorine resistance of single-species biofilms has been found to be highly related to the physiological state of bacterial cells. For example, when five strains isolated from simulated DWDS formed single-species biofilms, where bacteria formed spores or secreted EPS, the biofilms showed a greater chlorine resistance [9]. This agrees with another study that indicated that bacteria with the highest EPS production showed the strongest chlorine resistance [12]. Gram-positive bacteria from fresh water were more resistant to chlorine than Gram-negative [13].

Packman et al. [14] described how processes like chlorine disinfection can contribute to the persistence and dissemination of bacterial pathogens from biofilms. They found that the concentration of disinfectant in drinking water was not correlated with the abundance of bacteria within the biofilms and they challenged the standard practice of maintaining chlorine residual to inactivating water-borne pathogens, thus adding to a debate on chlorination practices that has existed for many years [15]. Fish et al. [16,17] showed that high-chlorine concentrations reduced biofilm cell concentrations in simulated DWDS, but selected for a distinct biofilm bacterial community and inorganic composition, which presented unique risks. In addition to that, there were several studies from which it is clear that chlorine residual could not prevent biofilm development [18,19,20], but could only impact on the quantity of bacteria and cause microbial community shifts. Our study sheds light on the impact of chlorine on multi-species biofilm formation and development under different flow conditions. Thus, we focus on measurements of several biofilm characteristics, such as biofilm thickness, density, and biofilm coverage as well as number, size and coverage of cell and EPS clumps both in water and on surfaces.

Here, we explore the impact of the chlorine disinfectant residual on the formation and development of multi-species biofilms in tap water. We aim to add evidence to the debate on whether disinfectant residuals act to reduce or promote biofilms. We studied biofilms grown in chlorinated and unchlorinated water in stagnant conditions and in a bioreactor under different turbulent flow regimes that are characteristic in main water pipes [21].

## 2. Materials and Methods

### 2.1. Experimental System and Conditions

Reactor slides were placed in two trays and left in water under stagnant conditions in an incubator. The trays (Retail Acrylics, Cardiff, UK) had dimensions of 50 cm by 50 cm, they were made of acrylic material (AT500L-D), and they were sterilized before use. One tray had 45 reactor slides in tap water and the other tray had 45 slides in tap water that had been allowed to sit for 24 h allowing the chlorine in water to decay [22,23], without affecting the indigenous microbial community. We endeavoured to set the chlorine concentration in the first tray of ‘chlorinated’ water at 1 mg/L at the onset of each day. To achieve that, we sampled drinking water from a tap at the Biotechnology Lab in the ARC (Advanced Research Centre building, University of Glasgow, Glasgow G11 6EW, UK). We measured the total chlorine concentration in raw water for 24 h in the incubator at 20 °C and in the reactor at 16.6 °C. On average, the concentration of total chlorine decayed to below 0.1 mg/L after 24 h (Tables S1 and S2 in Supplementary Material). When we added 600 μL of sodium hypochlorite solution (6–14% active chlorine, Supelco, Emplura, Darmstadt, Germany), the concentration rose to the desired 1 mg/L. Given that sodium hypochlorite solution was dosed once per day and subsequently decayed in our chlorinated water experiment, the chlorine concentration fluctuated between 0.53 and 1.03 mg/L (Table S3 in Supplementary Material). Chlorine measurements were conducted using the USEPA DPD Method 8167 and a colorimeter (DR 900 Hach, Loveland, CO, USA) [24].

The reactor with the slides used for our experiments was the Rotating Annular Reactor (RAR) from the BioSurface Technologies Corporation in United States (Bozeman, MT, USA). It was sterilized before use with 70% ethanol according to the company’s protocol. The reactor slides were of polycarbonate material (BST-503-PC). They had a height of 14.9 cm, a width of 1.2 cm, an area of 17.5 cm^2^ and a volume of 2.80 cm^3^. The polycarbonate material was chosen for the reactor slides as one of the plastic materials used in DWDS, which has neither rough nor corroded surface [25,26]. The reactor was operated in batch mode for all flow conditions as the goal was to maintain a well-controlled system for our experiments to promote the formation of biofilms on the reactor slides [27].

The incubated slides from the static culture were then moved to the reactor: 12 slides for each of the three flow regimes in chlorinated water and another 12 slides for each of the three flow regimes in chlorine-free water. In addition, 3 slides were used to characterize biofilms immediately after the incubation period for both experiments. This number of slides allowed us to have triplicates for all our surface measurements. The incubation period was seven days. We chose 24 h for the reactor experiments as a sufficient time for biofilms to adapt to each flow regime [21,28]. The incubation temperature was at 20 °C [29], which allowed biofilms to grow within a week [30,31]. The average temperature water in the reactor was 16.6 °C, which is a representative water temperature in the United Kingdom [11]. These temperatures were measured daily using a thermometer (Fisherbrand™ 10/30 Ground Joint Thermometer, Fisher Scientific, Loughborough, UK). The rotation speeds of the RAR for the three flow regimes were 217, 169 and 121 rpm, which correspond, respectively, to Reynolds numbers of 7500, 5900 and 4200 (Equation (1)), average speeds of 0.25, 0.20 and 0.13 ms^−1^, and shear stresses of 0.28, 0.18 and 0.08 Nm^−2^. These were calculated based on Taylor–Couette flow equations [32,33,34,35,36]. The Reynolds numbers show that all the regimes were turbulent.

Re = uD/v,(1)

In Equation (1), u is the velocity of the fluid, which in the case of the reactor is the product of the rotating speed of the reactor and the radius of the inner rotating cylinder. In the equation, D is two times the radius of the reactor annulus (the gap between the outer and inner reactor cylinders). Lasty, v is the kinematic viscosity of the fluid.

### 2.2. Biofilm Measurements

Gravimetric measurements were performed to characterise the thickness and density of biofilms on the reactor slides as described in an earlier study [37]. The measurements were taken after the end of each flow regime in the reactor. In brief, three slides were removed from the reactor, drained for five minutes at a vertical position, and weighed for the determination of the wet mass. Then, the slides were dried for 24 h at 65 °C in an oven (Carbolite Gero Ltd., Hope Valley, UK) and weighed again. After that, the dried biofilm was washed off the slides with distilled water and laboratory tissues. The clean slides were dried again for 24 h at 65 °C and then weighed. The dry mass was determined by the weight difference of the slides with and without the dried biofilm. After the calculations of the weight of the wet and dry biomass, and the clear weight of the reactor slides, we calculated the biofilm thickness and density. For these measurements, the Fisherbrand™ Analytical Balance MH-214 was used (Fisher Scientific, UK) with 0.0001 g precision.

Three slides were used for each microscopy measurement. Biofilms on the reactor slides were fixed with 0.5 mL of 4% paraformaldehyde where necessary [38]. The samples were firstly covered with 1 mL of 10 μg/mL Fluorescein Aleuria aurantia lectin (Vector laboratories, Peterborough, UK) for 10 min in the dark to stain the EPS [26,39]. Then, they were covered with 1 mL of 10 μg/mL 4′,6-diamidino-2-phenylindole (DAPI) (ThermoFisher Scientific, Loughborough, UK) for 20 min in the dark to stain the cells. Biofilm structures were visualised directly on the slides using the Evos FL Auto2 microscope (Invitrogen, Waltham, MA, USA; ThermoFisher Scientific, Loughborough, UK). The filters used were the FITC filter with excitation at 495 nm and emission at 525 nm for EPS visualisation and the DAPI filter with excitation at 358 nm and emission at 461 nm for total cells visualisation. The lens used was an Olympus UPlanSApo 40×/0.95. The analysis of our microscopy images was performed in Matlab. The original images were firstly converted to grey-scale images using the command “rgb2gray” and then to binary images using the command “im2bw” to separate the biomaterial from the background of the image. Then, the number of cell and EPS clumps was calculated using the command “bwlabel”. Also, the area of cell and EPS clumps was calculated using the command “regionprops”. The total average coverage surface area that the cell and EPS clumps occupied was finally calculated. After the surface areas were calculated, they were divided to the total surface to finally calculate the percentages of the coverage (%). Lastly, the relative frequency of the area of the cell and EPS clumps was calculated in Matlab using the functions cdf (cumulative distribution function) and ecdf (empirical cumulative distribution function).

### 2.3. Biomaterial Measurements

To count cells, three 5 mL samples were used from the bulk water of the reactor. We fixed the cells by adding 0.5 mL of 2% formaldehyde [40] and then we filtered the liquid samples on Whatman^®^ 0.2 μm membrane filters (Sigma-Aldrich, Irvine, UK). The filters were covered with 1 mL of 0.1% Triton X-100 solution (ThermoFisher Scientific, Loughborough, UK) to evenly disperse the cells before DAPI was applied. A solution of 1 mL of 10 μg/mL DAPI was used to stain the cells for 20 min in the dark. After that, the solution was filtered, and the membrane filters were dried and prepared for visualisation. Images were obtained for the cells on the membrane filters using the Evos FL Auto2 microscope. The lens used was an Olympus UPlanSApo 40×/0.95 and the filter used was the DAPI filter. The same measurements as on the slides were performed for the biomaterial (cells and EPS) in the water samples. For each case, three liquid samples of 10 mL each were analysed. The biomaterial from the water samples was processed as described in the cell count measurements in water (without the addition of the Triton solution). Again, the Fluorescein Aleuria aurantia lectin was used to stain the EPS in the water samples. In total, three calculations were made: the number of cell and EPS clumps, the area of cell and EPS clumps, and the relative frequency of clumps.

### 2.4. Statistical Analysis

There were three different analyses; one analysis was to compare biofilms in chlorine and non-chlorine experiments, the second one was to compare cells and EPS clumps in both experiments, and the third one was to compare biofilms in the different flow regimes. All measures were analysed in IBM SPSS Statistics program using one of the following tests: (i) the one-way ANOVA test in conjunction with the Tukey’s and Duncan–Waller’s tests, (ii) the Kruskal–Wallis by ranks test, (iii) the Jonckheere–Terpstra test and finally, (iv) the Pearson’s chi-squared test in conjunction with the Phi and Cramer’s test, depending on the fitness of the data under comparison to the assumptions of the statistical tests. All statistical calculations were based on the confidence level of 95%, which means that a *p* value lower than 0.05 was considered to be statistically significant. Where the data sets were normally distributed and there was homogeneity of variances, then significant differences between data were tested using the one-way ANOVA test in conjunction with the Post hoc Tukey’s and Duncan–Waller’s tests that would further validate the statistical result of one-way ANOVA. Where the data sets were not normally distributed, then the Kruskal–Wallis test was used, in which the variances of the populations should be equal across the samples. For the data in which the condition for the variances of the Kruskal–Wallis test was not met, the Jonckheere–Terpstra test was deployed, in which there should be a priori ordering of the populations. In the case in which the data sets did not validate the assumptions of any of the previous statistical tests, then the Pearson’s chi-squared test in conjunction with the Phi and Cramer’s statistical test were performed for large size samples for which there was the assumption that the data sets were consistent with a theoretical distribution.

## 3. Results

### 3.1. Biofilm Thickness and Density

**Figure 1:**
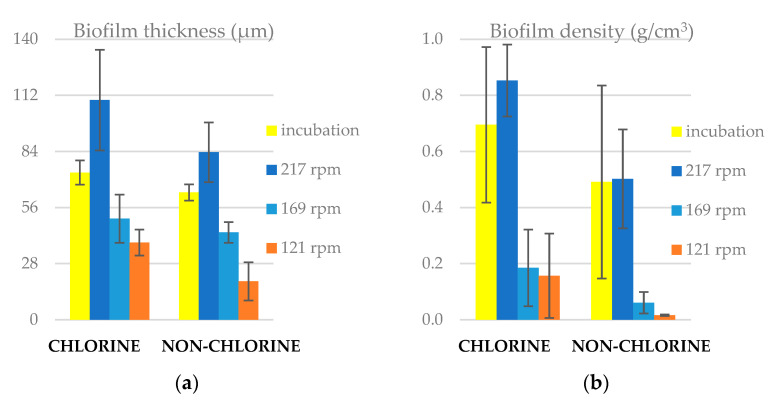
(**a**) Biofilm thickness and (**b**) biofilm density on reactor slides for chlorine and non-chlorine experiments under incubation and flow conditions.

The results showed that the biofilm thickness and density were higher in chlorine than in non-chlorine experiments under all conditions (Figure 1). During the 24 h, at the reactor rotating at 217 rpm, the biofilms thickness and density increased, confirming that the net movement was from the bulk water onto the slide surfaces. We also found that biofilm thickness and density decreased under the lower shear stress conditions (169 and 121 rpm). In all cases, the biofilms were found to be thin (ranging from 20 to 140 μm) as we expected for tap water biofilms [41,42]. From Table S4 in Supplementary Material, the statistical analysis indicated that the biofilm density was significantly higher (*p* < 0.05) in chlorine than non-chlorine experiments under all reactor conditions.

### 3.2. Number of Clumps on Slides

**Figure 2:**
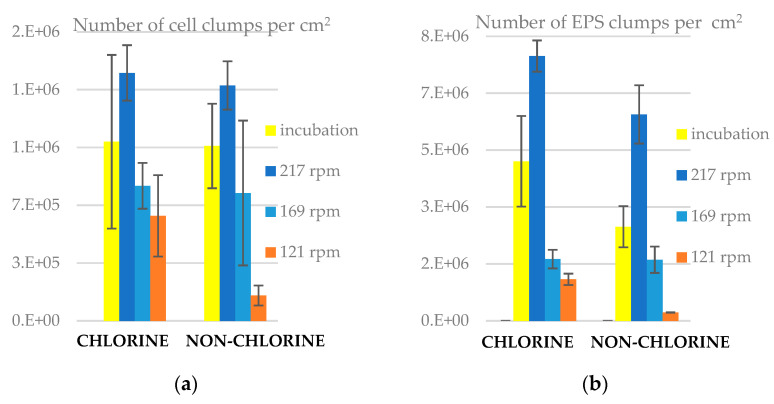
Number of clumps on slides for chlorine and non-chlorine experiments, (**a**) cell clumps and (**b**) EPS clumps. The error bars represent ± one standard deviation.

The biofilm on the slides did not comprise a uniform, homogeneous film, instead it was made up of patches, or clumps, of cells and EPS of differing sizes. Thus, part of characterizing the biofilm is to ascertain the number and distribution of clumps. The results in Figure 2 show that the number of EPS clumps was higher than that of cell clumps in both experiments. Thus, assuming the organic material identified as EPS was generated by bacteria, EPS clumps have separated from cells both in incubation and reactor stages of the experiment. After 24 h at 217 rpm, the number of clumps increased on the slides in both experiments. Under 169 and 121 rpm, the number of clumps decreased in both experiments, which again suggests that the net flux of biological material was from biofilms to the bulk at lower shear stresses. We found that the average number of clumps (both cells and EPS) per square centimetre was higher in chlorine than in non-chlorine experiments under all conditions, suggesting that the presence of chlorine promoted either the movement of clumps onto surfaces or the adhesion once they attached. The statistical analysis (Table S4 in Supplementary Material) indicated that the number of cell clumps was significantly higher (*p <* 0.05) in chlorine than in non-chlorine experiments at 217 rpm and at 121 rpm. The number of EPS clumps was significantly higher (*p <* 0.05) in chlorine than in non-chlorine experiments in incubation, 217 rpm and 121 rpm. Also, the number of EPS clumps was significantly higher (*p* < 0.05) than that of cell clumps in both chlorine and non-chlorine experiments.

### 3.3. Area of Clumps on Slides

**Figure 3:**
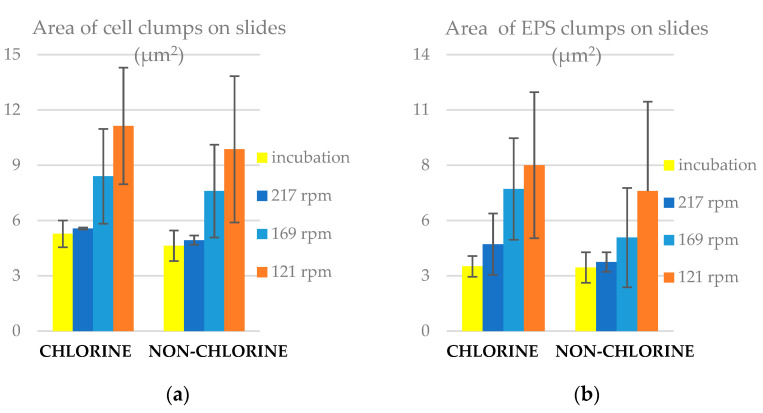
Area of clumps on slides for chlorine and non-chlorine experiments (**a**) cell clumps and (**b**) EPS clumps. The error bars represent ± one standard deviation.

The size of the clumps was characterized by their area (Figure 3). In both experiments, the size of cell clumps was greater than that of EPS clumps. We also found that the average size of clumps was higher in chlorine than in non-chlorine experiment. This suggests that it is not merely adhesion that is affected by chlorine, but also the growth or aggregation of cells in clumps is enhanced by its presence. The mean clump size on the post-incubation slides and the slides on the reactor turning at 217 rpm were lower than those on the slides in the slower rotating reactors, despite them having thicker biofilms and more clumps. The statistical analysis indicated that the area of cell clumps on the slides was significantly higher (*p* < 0.05) in chlorine than in non-chlorine experiments at 217 rpm (Table S4 in Supplementary Material). Also, the area of EPS clumps was significantly higher (*p* < 0.05) than that of cell clumps in both chlorine and non-chlorine experiments.

**Figure 4:**
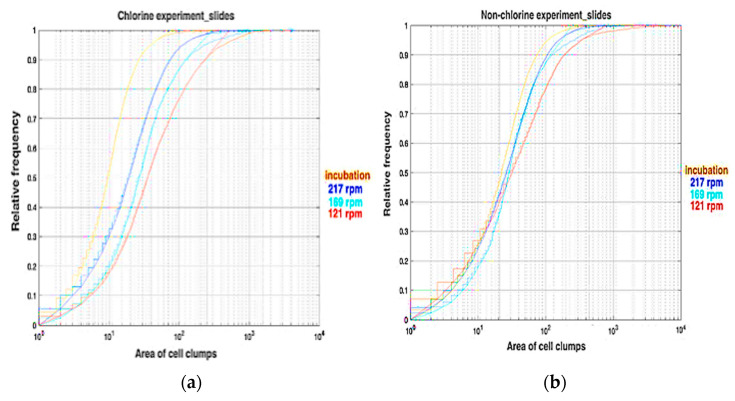
Relative frequency figures for the area of cell clumps (in square pixels) on slides for (**a**) chlorine and (**b**) non-chlorine experiments.

**Figure 5:**
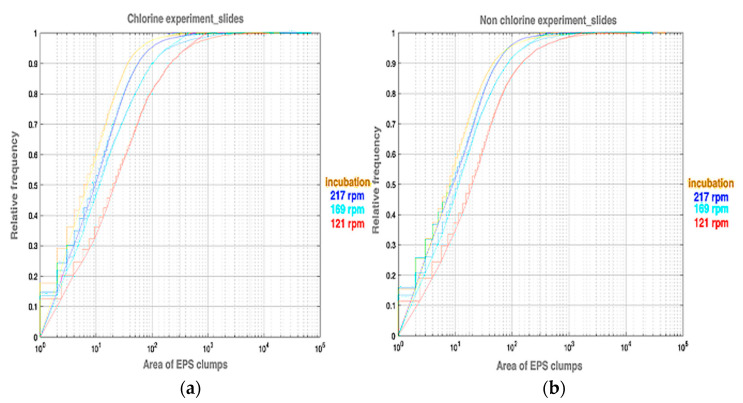
Relative frequency figures for the area of EPS clumps (in square pixels) for (**a**) chlorine experiment and (**b**) non-chlorine experiments.

To gain a better understanding of the distribution of clump sizes and how it is affected by the prevailing conditions, we plot the cumulative relative frequency of clump areas (cells: Figure 4 and EPS: Figure 5) for the chlorine and non-chlorine experiments. One pixel is equal to 0.1 μm, which means 1 pix^2^ is equal to 10^−2^ μm^2^. These figures show that the distributions of clump areas are most skewed towards smaller areas for the post incubation slides, followed by the ones at 217 rpm. The distribution for slides at 121 rpm is most skewed to towards higher clump areas. This is particularly pronounced for the chlorinated case. For the non-chlorinated case, there is little difference in the distributions at lower areas, but the distribution on the lowest rotation speed (121 rpm) slides is still skewed towards the higher areas. This might suggest that shear stress tends to expand clumps on the slides, but superimposed on this is the erosion, which can break up clumps and the deposition and adhesion of small clumps, which is enhanced by higher shear and chlorine concentrations. We fitted a log-normal cumulative probability distribution to each of the empirical cumulative frequency distributions, which confirmed a result from a previous study that the distribution of clump areas is well characterised by the lognormal distribution [43].

### 3.4. Coverage Area of Clumps on Slides

**Figure 6:**
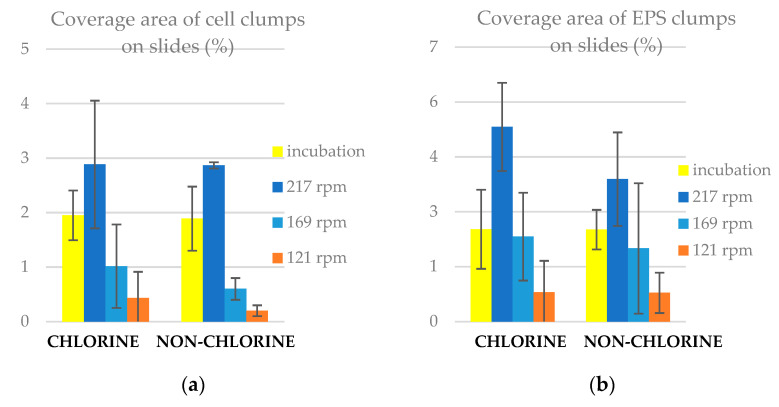
Coverage area of clumps on slides for chlorine and non-chlorine experiments (**a**) cell clumps and (**b**) EPS clumps. The error bars represent ± one standard deviation.

While several clumps per square micrometre found on the reactor slides might seem like a dense patch work, the coverage is shown in Figure 6 to be low. This is because these clumps are well distributed across the thickness of the biofilm. The coverage area of EPS clumps was higher than that of cells under all conditions, however, it should be noted that the coverage as a percentage of the slide surface was low for both. This reinforces the fact that these drinking water biofilms did not adhere to the classic view of homogenous films. We found that the coverage area of clumps (both cells and EPS) in percentages was higher in chlorine than in non-chlorine experiments under all conditions. The lowest coverage of clumps was for slides rotated at 169 and 121 rpm. This is despite the previous result where the mean clump sizes were greater in these lower shear conditions. Thus, the higher coverage that occurred immediately after cultivation and after 24 h in high shear 217 rpm conditions must be down to the higher absolute number of clumps. Overall, our results indicate that the presence of 1 mg/L of chlorine in tap water promoted the net movement of biomaterial onto the slides and its adhesion to form biofilms, especially under high shear stress.

### 3.5. Cell Concentration in Water

**Figure 7:**
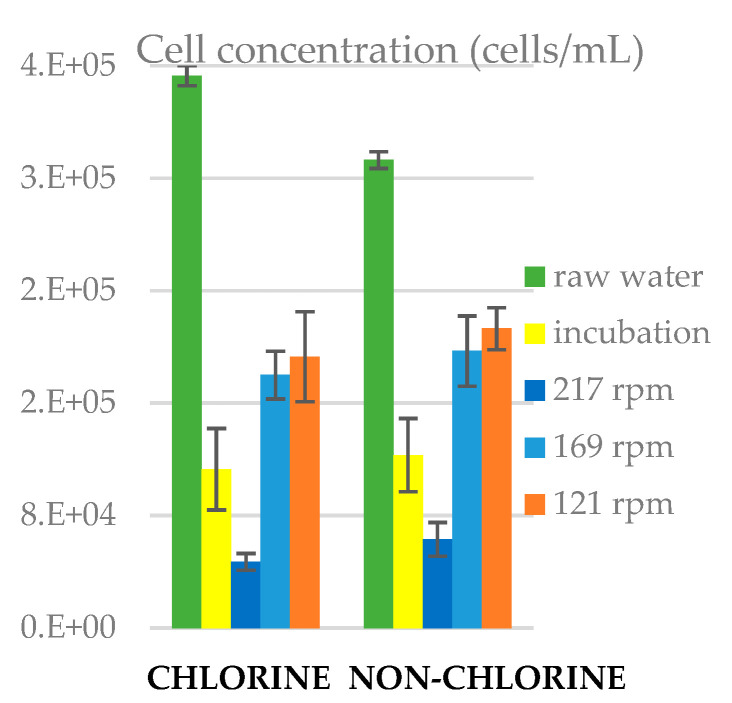
Cell concentration in water for chlorine and non-chlorine experiments under incubation and flow conditions. The error bars represent ± one standard deviation.

In the non-chlorinate experiment, the raw tap water sat for 24 h, while the chlorine concertation dropped, whereas for the chlorinated experiment the water was taken directly from the tap (Figure 7). This might explain the fact that the cell count prior to incubation is higher in the chlorinated than the non-chlorinated experiment. For both experiments, the cell count in the bulk water declined during incubation, suggesting that bacteria moved onto the slide surfaces. After 24 h in the reactor rotating at 217 rpm, the cell concentration further decreased. Thus, despite the high shear, which one might has anticipated at least initially eroding the biofilm built up during incubation, the net movement over the 24 h period appears to be from the bulk water onto the slides. For the experiments with lower rotation speeds, the net movement over the 24 h periods was from the slides back into the bulk liquid, as the cell counts were higher at the end of the period. We found that the cell concentration in water was significantly higher (*p <* 0.05) in non-chlorine than in chlorine experiments in incubation and at 121 rpm (Table S4 in Supplementary Material).

### 3.6. Number of Clumps in Water

**Figure 8:**
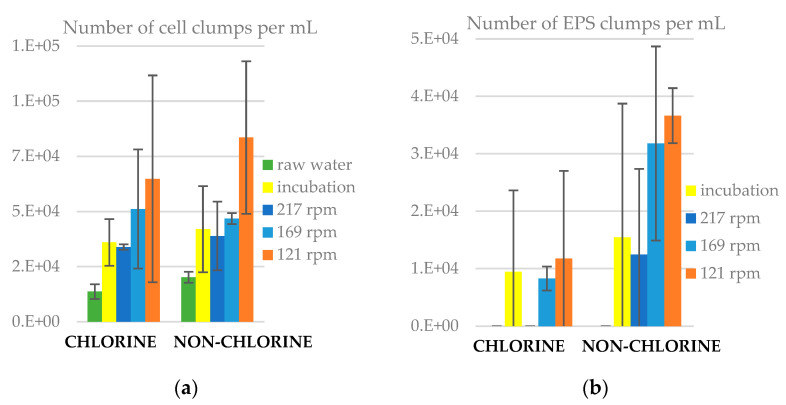
Number of clumps in liquid samples for chlorine and non-chlorine experiments, (**a**) cell clumps and (**b**) EPS clumps. The error bars represent ± one standard deviation.

In raw water there were clumps of cells, but no EPS clumps (Figure 8). After incubation, for both chlorinated and non-chlorinated cases, the cell clumps had approximately doubled, and many EPS clumps had appeared, still less than the cell clumps. Indeed, for both experiments, the number of cell clumps in the bulk water was higher than that of EPS clumps. Under 217 rpm, the number of cell clumps increased in water, but there were no EPS clumps. This shows that any erosion that occurred during the 24 h period did not remove EPS from the slides. Indeed, all the EPS that was in the post incubation bulk water was lost during the 24 h period at 217 rpm in chlorinated conditions and was significantly reduced without chlorine. At lower shear stresses (169 and 121 rpm), the number of cell and EPS clumps increased in water, which suggests again that over the 24 h, the biofilm erosion exceeded any deposition. From the statistical analysis (Table S4 in Supplementary Material), we found that the number of EPS clumps in water was significantly higher (*p <* 0.05) in non-chlorine than in chlorine experiments at 169 rpm. Also, the number of cell clumps was significantly higher (*p* < 0.05) than that of EPS clumps in both chlorine and non-chlorine experiments.

### 3.7. Area of Clumps in Water

**Figure 9:**
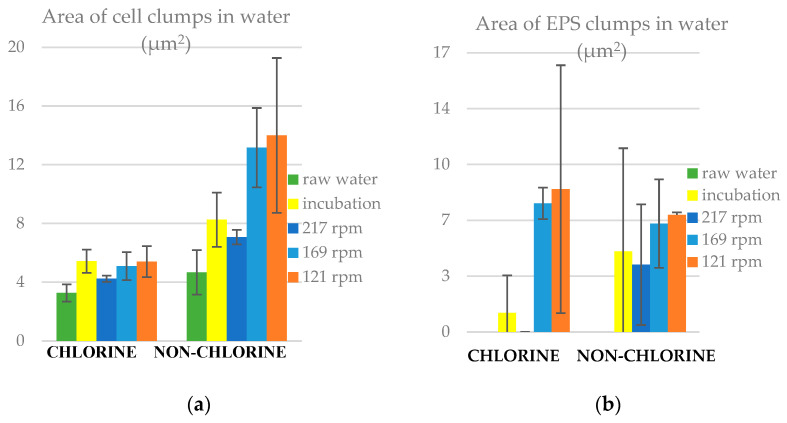
Area of clumps in liquid samples for chlorine and non-chlorine experiments, (**a**) cell clumps and (**b**) EPS clumps. The error bars represent ± one standard deviation.

The area of clumps (Figure 9) shows that during incubation both cell and EPS clumps, on average, grew, both in the chlorinated and non-chlorinated cases. The sizes of cell clumps were reduced under the high shear (217 rpm), suggesting that either larger clumps were preferentially laid down on to the slide surfaces or that smaller clumps were eroded from the slides. We found that the average area of clumps (both cells and EPS) was higher in non-chlorine than in chlorine experiments under all conditions. Again, this suggests that biofilm erosion was favoured under non-chlorine conditions. For a better understanding of the results, we calculated the relative frequency for the distribution of clumps and unlike for the slides, there was neither any discernible difference in the shape of the distribution nor a good fit to the lognormal distribution in both experiments (Figures S1 and S2 in Supplementary Material). Lastly, we found different interesting structures of biofilms from the EVOS microscopy images obtained (Figure S3 in Supplementary Material). Biofilms were found in both filamentous and patchy structures. From the statistical analysis (Table S4 in Supplementary Material), we found that the area of cell clumps in water was significantly higher (*p <* 0.05) in non-chlorine than in the chlorine experiment in all reactor conditions. Also, the area of cell clumps was significantly higher (*p* < 0.05) than that of EPS clumps in both chlorine and non-chlorine experiments.

## 4. Discussion

When polycarbonate slides sit in static tap water for a week, the clumps of cells expand and are initiated, presumably through a combination of aggregation and growth, and clumps of EPS also appear both attached to cells and separate. We found that the presence of chlorine in the water increases the prevalence and size of cell clumps. We then placed the slides into the inner wall of the anulus in an annular rotating bioreactor along with the water used during the incubation period. In three separate experiments, for both chlorinated water and non-chlorinated water, the reactor was set to rotate for 24 h at three different speeds 217, 169 and 121 rpm, which induced turbulent flows, with Reynolds numbers typical of pipe networks. The distribution of biomaterial on the slides and in the bulk water was affected differently by each of the regimes. Biofilms in chlorine experiments were shown to be more robust to shear stress as we indicated with our statistical results (Tables S5 and S6 in Supplementary Material).

Previous studies have investigated the interaction of chlorine with different biofilm clump sizes under different shear stress conditions. Tsai (2006) showed that there was no significant interaction of chlorine concentration and shear stress for particle numbers with 2–5, 5–15, 50–100 and >100 μm diameters. Instead, a significant interaction was found on particle numbers of 15–25 and 25–50 μm diameters [44]. Similarly, Behnke et al. in 2011 highlighted that the chlorine disinfection tolerance was highly dependent on the cluster size distribution of biofilms. It was shown that the disinfection efficacy was dependent on species composition; co-culture was advantageous to the survival of two bacterial species when grown as a biofilm or as clusters detached from biofilm but, surprisingly, there was lower disinfection tolerance when they were grown as a mixed planktonic culture [45]. In another study, Zhang et al. (2019) found that the disinfection effect on biofilm bacteria was dose dependent and species specific. Chlorine disinfection was shown to reduce bacterial numbers but had little effect on biofilms grown in annular reactors on cast iron coupons [46].

In a previous study, Kelly et al. in 2014 found that there was no significant relationship between the abundance of bacteria within the biofilms and the concentrations of disinfectant (monochloramine) in a region of a DWDS in Florida in USA, which showed that significant protection was provided to the bacteria by the biofilm matrix [47]. This is important as biofilms may consist of pathogenic bacteria posing serious risks to public health and it has been shown that under high shear stress conditions that typify DWDS biofilm formation are favoured [21,48]. Liu et al. in 2020 indicated that under turbulent flow they observed enhanced biofilm formation, with the highest biofilm/total cells ratio. Under the high shear stress conditions, EPS production was enhanced and resulted in pipe surface aggregation [49].

In our study, we did not observe the biofilm dynamics in real time and only observed the biofilm and water characteristics at the end of the 24 h period. Nonetheless, we can speculate on the mechanisms that might have affected the changes. It is reasonable to assume that the partitioning of material between the biofilms on the slides and the bulk water and the nature of the biofilms are affected by a combination of three main mechanisms, the relative importance of which are a function of the turbulence and shear stress. Firstly, biofilms will be eroded by the shear stress on the slide induced by the flow. Thus, single cells and clumps of varying sizes, less than or equal to those on the slides, of both cell and EPS, will be ripped from the slide and entrained into the bulk liquid. One would expect that the higher the flow rate, the higher the shear and thus, the greater the entrainment. Secondly, the processes of cell growth and EPS production will expand existing structures in both the bulk water and the slides. Thirdly, the cells and clumps of biomaterial in the flow will be advected in the flow and, ignoring the motility of cells for the moment, will be subject to molecular diffusion and dispersion due to the turbulence, which will deposit particles onto the slides. A fourth, more speculative process is the possibility that the properties of the biomaterial change under high turbulence to become more adhesive.

In our case, when we applied the highest shear at 217 rpm, we did not see an increase in the entrained material over the 24 h period. Indeed, all the EPS moved from the bulk liquid onto the slides. It may be that there is an initial entrainment event that we missed, but over the 24 h period, deposition exceeded entrainment. The fact that this did not happen at lower shears suggests that the deposition is positively correlated with turbulence intensity. This has been observed and indeed is the basis of several mathematical models of deposition [50,51].

Overall, this research aims to highlight to the water industry to look beyond the traditional practice and regulation of chlorine disinfection in water treatment and inform the existing disinfectant residual strategies in order to ensure safe drinking water. Future research is needed to investigate the role of chlorine disinfection residuals in biofilms and in water as recent studies show that they are associated with systematic impacts on the structure and functional potential of the drinking water microbiome [52], a selection of opportunistic pathogens [53], higher proportional abundance of deleterious microbes, such as mycobacteria, nitrifiers, and corrosion causing bacteria in biofilms [54], and finally, the presence of antibiotic resistance genes in the water [55].

Our main finding in this study supports that chlorine disinfectant residuals promote tap water biofilms on surfaces. This is significant, as the biofilms forming on the walls of water pipes via the EPS might be home for opportunistic pathogenic bacteria, which can result in serious human health risks. Previous studies have investigated specific species biofilms [9,56,57]. Our focus was on multi-species biofilms that can form from bacteria that live in our tap water systems. Further research on chlorine disinfectant strategies in DWDS is needed to ensure safe drinking water free of biofilm clumps and pathogens remains.

## Data Availability

The data presented in this study are available on request from the corresponding author.

## Supplementary Materials

The following supporting information can be downloaded at: https://www.mdpi.com/article/10.3390/microorganisms11112680/s1, Table S1. Concentration of total chlorine in preliminary experiment in raw water; Table S2. Concentration of total chlorine in preliminary experiment in raw water with the addition of sodium hypochlorite; Table S3 Concentration of total chlorine in chlorine experiment in the incubator and in the reactor; Figure S1. Relative frequency figures for the area of cell clumps (in square pixels) in water for (a) chlorine experiment and (b) non-chlorine experiments; Figure S2. Relative frequency figures for the area of EPS clumps (in pixels) in water for (a) chlorine and (b) non-chlorine experiments; Figure S3. Biofilm (EPS in green colour and cells in purple colour) images from EVOS FL Auto 2 cell imaging system using a 100X oil immersion objective lens. These images were selected randomly to indicate (a) filamentous structures and (b) round/patchy structures found in biofilms; Table S4. Statistical results for biomass measures of the same flow regime between chlorine and non-chlorine experiment: x—significant differences were found (*p* < 0.05); Table S5. Statistical results for biomass between the different flow regimes in chlorine experiment: 1—incubation, 2—217 rpm, 3—169 rpm, 4—121 rpm, 5—raw water, x—significant differences were found (*p* < 0.05); Table S6. Statistical results for biomass between the different flow regimes in non-chlorine experiment: 1—incubation, 2—217 rpm, 3—169 rpm, 4—121 rpm, 5—raw water, x—significant differences were found (*p* < 0.05).
