# Supplementary material for: The Role of Chlorine in the Formation and Development of Tap Water Biofilms under Different Flow Regimes
Source: Microorganisms. 2023 Oct 31;11(11):2680. doi: 10.3390/microorganisms11112680 (PMC10673482; doi:10.3390/microorganisms11112680)
Supplement: Supplementary file 1 [file microorganisms-11-02680-s001.zip › microorganisms-2674507-supplementary.pdf]

Supplementary Material

The role of chlorine in the formation and development of tap water biofilms under different flow regimes

Erifyli Tsagkari [1*], William Sloan [2]

Table S1. Concentration of total chlorine in preliminary experiment in raw water.

Concentration of total chlorine (mg/L)	reactor				
	hours	incubation	217 rpm	169 rpm	121 rpm
preliminary experiment/raw water	0	0.25	0.23	0.23	0.24
	2	0.1	0.11	0.1	0.12
	4	0.09	0.07	0.09	0.08
	6	0.06	0.05	0.05	0.07
	8	0	0	0	0
	24	0	0	0	0
average		0.08	0.08	0.08	0.09
st dev		0.092	0.086	0.086	0.089

Table S2. Concentration of total chlorine in preliminary experiment in raw water with the addition of sodium hypochlorite.

Concentration total chlorine (mg/L)	reactor				
	hours	incubation	217 rpm	169 rpm	121 rpm
preliminary experiment/add sodium hypochlorite	0	1.03	1.01	1	1.01
	2	0.83	0.74	0.89	0.82
	4	0.62	0.61	0.73	0.73
	6	0.61	0.6	0.64	0.66
	8	0.56	0.54	0.51	0.55
	24	0.43	0.41	0.46	0.51
average		0.68	0.65	0.71	0.71
st dev		0.215	0.206	0.212	0.185

Table S3 Concentration of total chlorine in chlorine experiment in the incubator and in the reactor.

Concentration total chlorine	chlorine experiment						
(mg/L)	Days	0 hour	24 hours			average	st dev
incubation	1	1.02	0.63	0.63	0.62	0.63	0.01
	2	1.01	0.59	0.59	0.59	0.59	0.00
	3	1.01	0.61	0.6	0.61	0.61	0.01
	4	1.03	0.63	0.62	0.61	0.62	0.01
	5	1.01	0.62	0.62	0.62	0.62	0.00
	6	1.02	0.65	0.64	0.65	0.65	0.01
	7	1.02	0.59	0.58	0.58	0.58	0.01
reactor 217 rpm	8	1.01	0.58	0.58	0.58	0.58	0.00
reactor 169 rpm	9	1.02	0.62	0.62	0.64	0.63	0.01
reactor 121 rpm	10	1.01	0.54	0.52	0.54	0.53	0.01

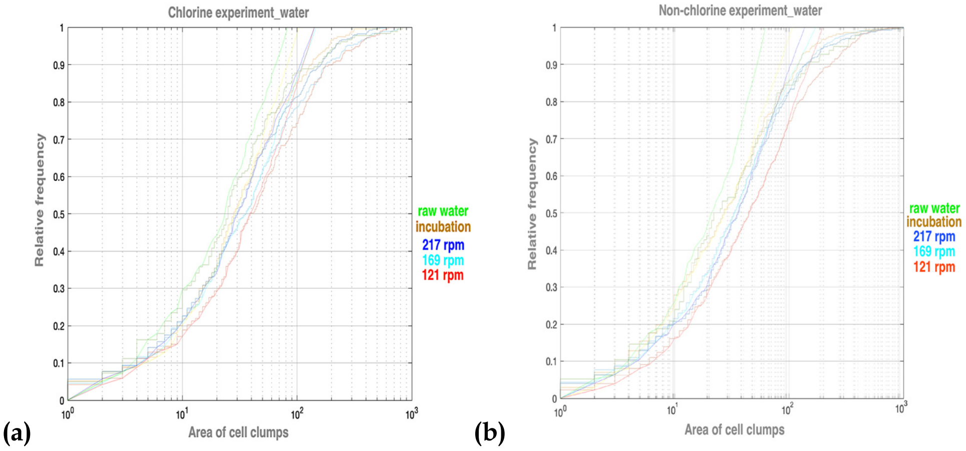

Figure S1. Relative frequency figures for the area of cell clumps (in square pixels) in water for (a) chlorine experiment and (b) non-chlorine experiments.

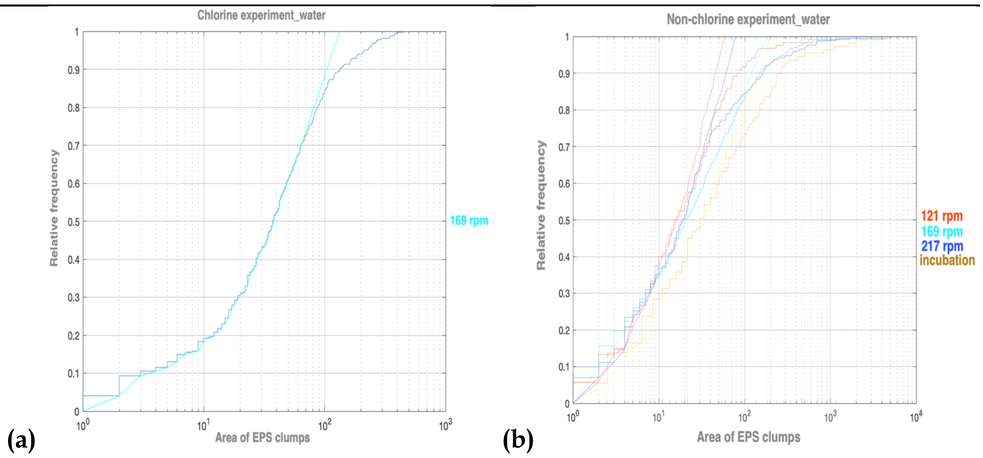

Figure S2. Relative frequency figures for the area of EPS clumps (in pixels) in water for (a) chlorine and (b) non-chlorine experiments.

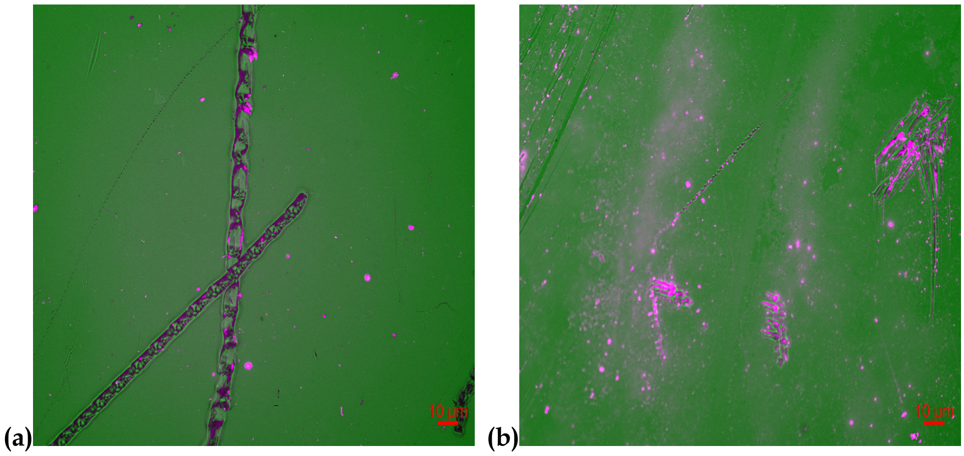

Figure S3. Biofilm (EPS in green colour and cells in purple colour) images from EVOS FL Auto 2 cell imaging system using a 100X oil immersion objective lens. These images were selected randomly to indicate (a) filamentous structures and (b) round/patchy structures found in biofilms.

Table S4. Statistical results for biomass measures of the same flow regime between chlorine and non-chlorine experiment: x- significant differences were found ($P < 0.05$).

Measures/ Flow regimes	Number of cell clumps/ water	Number of EPS clumps/ water	Area of cell clumps/ water	Area of EPS clumps/ water	Cell/ mL	Biofilm thickness	Biofilm density	Number of cell clumps/ slides	Number of EPS clumps/ slides	Area of cell clumps/ slides	Area of EPS clumps/ slides	Coverage area of cell clumps/ slides	Coverage area of EPS clumps/ slides
raw water													
incubation					x	x			x				
217 rpm			x		x		x		x	x			
169 rpm		x	x				x						
121 rpm			x		x		x	x	x				

Table S5. Statistical results for biomass between the different flow regimes in chlorine experiment: 1- incubation, 2- 217 rpm, 3- 169 rpm, 4- 121 rpm, 5- raw water, x- significant differences were found ($P < 0.05$).

Measures/ Flow-regimes	Number of cell clumps/ water	Number of EPS clumps/ water	Area of cell clumps/ water	Area of EPS clumps/ water	Cells/ mL	Biofilm thickness	Biofilm density	Number of cell clumps/ slides	Number of EPS clumps/ slides	Area of cell clumps/ slides	Area of EPS clumps/ slides	Coverage area of cell clumps/ slides	Coverage area of EPS clumps/ slides
1-2			x			x			x				x
1-3				x		x	x		x		x		x
1-4				x		x	x		x	x	x	x	x
1-5	x		x	x	x								
2-3		x		x	x	x	x	x	x				x
2-4							x	x	x		x	x	x
2-5					x								
3-4									x				
3-5													
4-5	x		x		x								

Table S6. Statistical results for biomass between the different flow regimes in non-chlorine experiment:1- incubation, 2- 217 rpm, 3- 169 rpm, 4- 121 rpm, 5- raw water, x- significant differences were found ($P < 0.05$).

Measures/ Flow regimes	Number of cell clumps/ water	Number of EPS clumps/ water	Area of cell clumps/ water	Area of EPS clumps/ water	Cell/ mL	Biofilm thickness	Biofilm density	Number of cell clumps/ slides	Number of EPS clumps/ slides	Area of cell clumps/ slides	Area of EPS clumps/ slides	Coverage area of cell clumps/ slides	Coverage area of EPS clumps/ slides
1-2						x			x			x	x
1-3			x			x			x	x		x	
1-4			x			x	x	x	x	x		x	x
1-5	x		x										
2-3			x			x	x		x	x		x	
2-4						x	x	x	x			x	
2-5	x	x	x		x								
3-4						x		x	x			x	
3-5	x	x			x								
4-5	x	x	x	x	x								
